# Scale-up of integrated malaria vector control: lessons from Malawi

**DOI:** 10.2471/BLT.15.154245

**Published:** 2015-04-21

**Authors:** Emmanuel Chanda, Themba Mzilahowa, John Chipwanya, Doreen Ali, Peter Troell, Wilfred Dodoli, Abraham P Mnzava, Birkinesh Ameneshewa, John Gimnig

**Affiliations:** a Malaria Vector Control Consultant, 11 Granite Street, Off Kamwala South Road, Plot 33421/917, PO Box 30146, Kamwala South, 10101 Lusaka, Zambia.; b Malaria Alert Centre, Chichiri, Blantyre, Malawi.; c Ministry of Health, National Malaria Control Programme, Lilongwe, Malawi.; d Centers for Disease Control and Prevention, Atlanta, Georgia, United States of America (USA).; e World Health Organization, Country Office, Lilongwe, Malawi.; f Global Malaria Programme, World Health Organization, Geneva, Switzerland.; g World Health Organization, Regional Office for Africa, Brazzaville, Congo.

## Abstract

**Problem:**

Indoor residual spraying and long-lasting insecticidal nets (LLINs) are key tools for malaria vector control. Malawi has struggled to scale up indoor residual spraying and to improve LLIN coverage and usage.

**Approach:**

In 2002, the Malawian National Malaria Control Programme developed guidelines for insecticide treated net distribution to reach the strategic target of at least 60% coverage of households with an LLIN. By 2005, the target coverage was 80% of households and the Global Fund financed the scale-up. The US President’s Malaria Initiative funded the indoor residual spraying intervention.

**Local setting:**

Malawi’s entire population is considered to be at risk of malaria. Poor vector control, insecticide resistance in malaria vectors and insufficient technical and financial support have exacerbated the malaria burden.

**Relevant changes:**

Between 2002 and 2012, 18 248 206 LLINs had been distributed. The coverage of at least one LLIN per household increased from 27% (3689/13 664) to 58% (1974/3404). Indoor residual spraying coverage increased from 28 227 to 653 592 structures between 2007 and 2011. However, vector resistance prompted a switch from pyrethroids to organophosphates for indoor residual spraying, which increased the cost and operations needed to be cut back from seven to one district. Malaria cases increased from 2 853 315 in 2002 to 6 748 535 in 2010, and thereafter dropped to 4 922 596 in 2012.

**Lessons learnt:**

A single intervention-based approach for vector control may have suboptimal impact. Well-coordinated integrated vector management may offer greater benefits. A resistance management plan is essential for effective and sustainable vector control.

## Résumé

**Problème:**

La pulvérisation d’insecticides à effet rémanent à l’intérieur des habitations et l'utilisation de moustiquaires à imprégnation durable (MID) sont des mesures clés dans la lutte antivectorielle contre le paludisme. Mais le Malawi a eu du mal à intensifier les interventions de pulvérisation intradomiciliaire d'insecticides et à améliorer la protection par des MID et leur utilisation.

**Approche:**

En 2002, le programme national de lutte contre le paludisme au Malawi a conçu des directives pour distribuer des moustiquaires imprégnées d'insecticides en vue d'atteindre l'objectif stratégique d'au moins 60% des ménages utilisant une MID. En 2005, la couverture ciblée est passée à 80% des ménages, et le Fonds mondial a financé l'intensification de la campagne. Les interventions de pulvérisation intradomiciliaire d'insecticides à effet rémanent ont été financées par l'Initiative du Président des États-Unis contre le paludisme.

**Environnement local:**

On considère que l'intégralité de la population du Malawi est exposée au risque palustre. Mais une lutte antivectorielle déficiente, le développement d'une résistance aux insecticides chez les vecteurs du paludisme et un soutien technique et financier insuffisant ont aggravé la charge du paludisme.

**Changements significatifs:**

Entre 2002 et 2012, 18 248 206 MID ont été distribuées. L'utilisation d'au moins une MID dans chaque ménage est passée de 27% (3 689/13 664) à 58% (1 974/3 404). La couverture par pulvérisation intradomiciliaire à effet rémanent est passée de 28 227 à 653 592 structures entre 2007 et 2011. Néanmoins, la résistance des vecteurs aux insecticides a nécessité d'abandonner les pyréthrinoïdes au profit des organophosphorés pour les pulvérisations intradomiciliaires, ce qui a majoré les coûts et nécessité de limiter les interventions à un seul district, sur les sept initialement prévus. Les cas de paludisme ont augmenté de 2 853 315 en 2002 à 6 748 535 en 2010 avant de chuter à 4 922 596 en 2012.

**Leçons tirées:**

En matière de lutte antivectorielle, une approche fondée sur un seul type d'intervention peut avoir des effets sous-optimaux. Une gestion antivectorielle intégrée correctement coordonnée pourrait être plus bénéfique. Un plan de gestion de la résistance est essentiel pour une lutte antivectorielle efficace et durable.

## Resumen

**Situación:**

La fumigación de interiores con acción residual y los mosquiteros tratados con insecticidas de larga duración (LLIN, por sus siglas en inglés) son herramientas importantes para el control de vectores de la malaria. Malawi ha luchado por aumentar la fumigación de interiores con acción residual y por mejorar la cobertura y uso de los LLIN.

**Enfoque:**

En 2002, el Programa de Control Nacional contra la Malaria de Malawi desarrolló directrices para la distribución de mosquiteros tratados con insecticidas con el fin de alcanzar el objetivo estratégico de que como mínimo el 60% de los hogares dispusieran de un LLIN. En 2005, la cobertura objetivo era un 80% de los hogares y el Fondo Mundial financió la ampliación. La Iniciativa del Presidente de los Estados Unidos de América contra la Malaria financió la intervención de la fumigación de interiores con acción residual.

**Marco regional:**

Se considera que toda la población de Malawi corre el riesgo de sufrir malaria. Los escasos controles de vectores, la resistencia de los insecticidas en los vectores de la malaria y el apoyo técnico y financiero insuficiente han multiplicado las tasas de malaria.

**Cambios importantes:**

Entre 2002 y 2012, se han distribuido 18 248 206 LLIN. La cobertura de al menos un LLIN por hogar ha aumentado de un 27% (3 689/13 664) a un 58% (1 974/3 404). La cobertura de fumigación de interiores con acción residual ha aumentado de 28 227 a 653 592 estructuras entre 2007 y 2011. No obstante, la resistencia de vectores ha acelerado un cambio de piretroides a organofosfatos para la fumigación de interiores con acción residual, lo que ha aumentado el coste y las operaciones necesarias para reducir de siete distritos a uno. Los casos de malaria aumentaron de 2 853 315 en 2002 a 6 748 535 en 2010, y luego cayeron a 4 922 596 en 2012.

**Lecciones aprendidas:**

Un enfoque basado en una única intervención para el control de vectores puede tener un impacto subóptimo. Una gestión integrada de vectores bien coordinada puede ofrecer mayores beneficios. Es fundamental desarrollar un plan de gestión de resistencia para lograr un control de vectores eficaz y sostenible.

## ملخص

**المشكلة:**

تتمثل الوسائل الرئيسية لمكافحة ناقلات مرض الملاريا في رش الأماكن المغلقة بمبيدات حشرية تدوم طويلاً واستعمال الناموسيات المعالجة بمبيدات حشرية مديدة المفعول (LLIN). ناضلت ملاوي من أجل زيادة معدل رش الأماكن المغلقة بمبيدات حشرية تدوم طويلاً لتحسين مدى توفير الناموسيات من نوع LLIN واستعمالها.

**الأسلوب:**

تم العمل في عام 2002 بالبرنامج الوطني لمكافحة الملاريا في ملاوي على وضع مبادئ توجيهية لتوزيع الناموسية المعالجة بمبيد حشري سعيًا إلى تحقيق الهدف الاستراتيجي الرامي إلى توفير الناموسيات بما يغطي 60‏% على الأقل من الدور. وبلغت نسبة التغطية 80% من الدُور مع حلول عام 2005، وقد قدّم الصندوق العالمي التمويل اللازم لرفع تلك النسبة. ووفرت مبادرة الرئيس الأمريكي لمكافحة الملاريا التمويل اللازم لتنفيذ التدخل المتعلق برش الأماكن المغلقة بمبيدات حشرية تدوم طويلاً.

**المواقع المحلية:**

تم اعتبار سكان ملاوي بأكملهم من المعرضين لخطر الإصابة بمرض الملاريا، فقد أدى انخفاض مستوى مكافحة ناقلات المرض، ومقاومة ناقلات مرض الملاريا للمبيدات الحشرية، ونقص الدعم المالي والتقني إلى تفاقم عبء مرض الملاريا.

**التغيرات ذات الصلة:**

تم توزيع 18,248,206 من الناموسيات من نوع LLIN في الفترة بين عامي 2002 و2012. وقد ارتفع مستوى التغطية التي تهدف إلى توفير ناموسية واحدة على الأقل من نوع LLIN لكل منزل من 27‏% (3689/13,664) ليصل 58‏% (1974/3404). كما ارتفع مستوى التغطية التي تهدف إلى رش الأماكن المغلقة بمبيدات حشرية تدوم طويلاً من 28,227 منشأة ليصل إلى 653,592 منشأة في الفترة بين عامي 2007 و2011. وبالرغم من ذلك، كانت مقاومة ناقلات المرض للمبيدات دافعًا للتحول من استخدام مادة بيريثرويد إلى المركبات الفوسفاتية العضوية فيما يتعلق برش الأماكن المغلقة بمبيدات حشرية تدوم طويلاً، مما أدى إلى ارتفاع التكلفة وزيادة الإجراءات الواجب تقليلها من سبع مناطق ليقتصر على منطقة واحدة. وزادت حالات الإصابة بمرض الملاريا من 2,853,315 حالة في عام 2002 لتصل إلى 6,748,535 حالة في عام 2010، ومنذ ذلك الحين انخفض عدد الحالات ليصل إلى 4,922,596 حالة في عام 2012.

**الدروس المستفادة:**

إن اتباع أسلوب يعتمد على تدخل واحد لمكافحة ناقلات المرض قد يكون أثره دون الحد الأمثل. وقد تحقق الإدارة المتعلقة بناقلات المرض والتي تتسم بالتكامل وحسن التنسيق المزيد من المنافع. كما أن توفر خطة لإدارة مقاومة المبيدات أمر ضروري لتحقيق المكافحة الفعالة والمستمرة لناقلات المرض.

## 摘要

**问题:**

室内滞留喷剂和长效驱虫蚊帐 (LLIN) 是控制疟疾媒介的重要工具。马拉维在推广室内滞留喷剂和提高长效驱虫蚊帐 (LLIN) 覆盖面和使用率方面困难重重。

**方法:**

2002 年，马拉维全国疟疾防治方案制定了一份指南，指导分发经过杀虫剂处理的蚊帐，以实现至少 60% 的住户拥有长效驱虫蚊帐 (LLIN) 的战略目标。到 2005 年，覆盖率目标为 80% 的住户，并且推广资金由全球基金会出资。 室内滞留喷剂干预由美国总统防治疟疾行动计划出资。

**当地状况:**

马拉维全体人口均面临疟疾的威胁。媒介控制水平差、疟疾媒介的抗药性以及技术和财政支持不足使得疟疾问题雪上加霜。

**相关变化:**

2002 年到 2012 之间，共分发了 18,248,206 件长效驱虫蚊帐 (LLIN)。每户至少一件长效驱虫蚊帐 (LLIN) 的覆盖率从 27% (3689/13 664) 增加到 58% (1974/3404)。2007 年到 2011 年之间，室内滞留喷剂覆盖率从 28, 227 户增加到 653, 592 户。但是，由于媒介的抗药性，必须使用有机磷酸酯类杀虫剂代替拟除虫菊酯进行室内滞留喷洒，这使得原计划用于 7 个地区的成本和运营减少至只能用于 1 个地区。从 2002 年到 2010 年，疟疾病例从 2,853,315 例增加到 6,748,535 例，然后，在 2012 年，降至 4,922,596 例。

**经验教训:**

单一的干预式媒介控制方法效果可能并不理想。相互协调的综合性媒介管理可能会取得更好的效果。抗药性管理计划是有效且可持续媒介控制的重中之重。

## Резюме

**Проблема:**

Остаточное распыление инсектицидов в помещении и инсектицидные сетки длительного действия (ИСДД) являются основными способами борьбы с переносчиками малярии. В Малави были предприняты упорные попытки расширить масштаб применения остаточного распыления инсектицидов и улучшить охват населения ИССД и показатели их использования.

**Подход:**

В 2002 году Национальной программой Малави по борьбе с малярией были разработаны рекомендации по распространению сеток, обработанных инсектицидами, для достижения стратегической цели — как минимум 60%-го охвата домашних хозяйств ИСДД. К 2005 году программа ставила целью уже 80%-й охват и Глобальный фонд профинансировал такое расширение. Инициатива президента США по борьбе с малярией выделила средства на остаточное распыление инсектицидов в помещениях.

**Местные условия:**

Считается, что все население Малави входит в группу риска в отношении заболевания малярией. По причине неудовлетворительных результатов борьбы с переносчиками, устойчивости переносчиков малярии к инсектицидам и недостаточной технической и финансовой поддержки бремя малярии усилилось.

**Осуществленные перемены:**

В период с 2002 по 2012 год было распространено 18 248 206 ИСДД. Охват населения (по меньшей мере одной ИСДД на домашнее хозяйство) вырос с 27% (3689 из 13 664 домохозяйств) до 58% (1974 из 3404 домохозяйств). Число опрыскиваемых помещений возросло с 28 227 до 653 592 в период с 2007 по 2011 год. Однако устойчивость, выработанная переносчиками, обусловила необходимость использовать для остаточного распыления органофосфаты вместо пиретроидов, из-за чего увеличилась стоимость обработки и охват соответствующими операциями пришлось сократить с семи округов до одного. Число случаев заболевания малярией выросло с 2 853 315 в 2002 году до 6 748 535 в 2010 году. Затем это количество сократилось до 4 922 596 случаев в 2012 году.

**Выводы:**

Подход к борьбе с переносчиками заболевания, основанный на одном способе вмешательства, возможно, не позволил добиться оптимального эффекта. Большую пользу могут принести качественно скоординированные мероприятия по комплексной борьбе с переносчиками заболевания. Для эффективной и устойчивой борьбы с переносчиками заболевания необходим план противодействия устойчивости к инсектицидам.

## Introduction

Malaria remains an important cause of morbidity and mortality. Approximately 214 million malaria cases and 438 000 malaria-related deaths were reported globally in 2015. The highest burden is seen in sub-Saharan Africa, where over 88% of all malaria episodes and 90% of all malaria-related deaths occur.^1^ The huge malaria burden in sub-Saharan Africa has been partly attributed to the presence of efficient vectors that maintain high levels of transmission.^2^ For vector control, the World Health Organization (WHO) recommends integrated vector management.^3^ Important pillars of the integrated vector management framework are insecticide treated nets and indoor residual spraying (hereafter referred to as indoor spraying).^4^

In Malawi, malaria control includes early definitive diagnosis of febrile cases by microscopy or use of a rapid diagnostic test, treatment with artemisinin-based combination therapy and the use of long-lasting insecticidal nets (LLINs), indoor spraying and intermittent preventive treatment.^5^ This paper describes the approaches used to scale up vector control in Malawi, the challenges encountered, the lessons learnt from this experience, and how these lessons have informed vector control efforts.

## Local setting

Malaria is endemic across Malawi and the entire population is considered to be at risk of the disease.^5^^,^^6^ Transmission of the causative parasite is high – more than one case per 1000 residents – and perennial with substantial seasonal variation in transmission intensity.^5^ The main vectors are *Anopheles gambiae* sensu stricto, *A. arabiensis* and *A. funestus*.^7^

To improve malaria control in the country, the Malawian National Malaria Control Programme was established in 1984. The programme’s tasks are to set policies, institute strategies, coordinate, monitor and evaluate activities, provide technical assistance and mobilize resources for malaria control. The first two national malaria strategic plans (1984–1989 and 1990–1994) that the programme developed had a focus on effective disease management.^8^ Since 1997, the programme has been distributing insecticide treated nets to children younger than five years and pregnant women countrywide.^5^ The vector control interventions of the programme remained small in scope and scale until 2007 when LLINs were delivered on a large scale. In 2010, the programme scaled up the coverage of indoor spraying.

## Approach

### Policy and guidance

To facilitate reaching the target of 60% household coverage by 2005, set in the 2000–2005 strategic plan, the programme developed guidelines in 2002 for distribution and use of insecticide treated nets. In the following strategic plans, the target was raised to 80% household coverage by 2010^8^ and then further raised to 90% of households owning at least one net and net usage to 80% by 2015. Integrated vector management and indoor spraying were introduced in the 2006–2010 strategic plan but were only consolidated during the 2011–2015 strategic plan.^9^ Programmatic implementation of integrated vector management began in 2007. In the same year, the Global Fund to Fight AIDS, Tuberculosis and Malaria financed the scale-up of LLINs and the US President’s Malaria Initiative funded the intervention of indoor spraying to reduce the burden of the disease by at least 50% by 2010 and 75% by 2015. The programme only uses the WHO prequalified insecticides for indoor spraying and prequalified net types of at least 100 denier.

A review of the programme in 2010 recommended that the strategy for 2011–2015 should emphasize an integrated vector management strategy, including LLINs, indoor spraying and approaches for management of larval sources and insecticide resistance.^9^

### Delivery mechanisms

The programme ran time-limited, intermittent mass distribution campaigns. Mass campaigns were conducted every three years and aimed at achieving coverage of one net for every 1.8 people.^9^ Planning and implementation of campaigns involved trained community health workers, government sister ministries and departments, nongovernmental organizations, private sector and civil societies. Before the campaign, communities were identified and households within these communities were registered and household members counted. Householders received education on how to hang, use and maintain the nets. The campaign used both door-to-door and centralized distribution approaches. If needed, mop-up campaigns followed each mass distribution. Nets were also distributed during the provision of routine health services, particularly to pregnant women and mothers of newborns who were attending clinics associated with the Expanded Programme on Immunization or antenatal care.

Using the pyrethroid lambda-cyhalothrin, the programme piloted indoor spraying in the Nkhotakota district in 2007. The indoor spraying was later expanded to seven districts with the plan of scaling up to 12 of the 28 Malawian districts by 2015.^10^ The programme aimed to ensure coverage of at least 85% of all structures in targeted districts. Entomological surveillance and resistance monitoring were done to inform decisions on vector control taken by the programme.^11^^,^^12^

### Community sensitization and mobilization

During World Malaria Day, the malaria commemoration week and the mass distribution campaigns, the programme conducted public awareness and social mobilization campaigns to create awareness, promote behavioural change and community participation and engagement. The mass media and interpersonal communication channels were used to disseminate advocacy, behavioural change and information and education communications. To ensure that the materials for both electronic and print media addressed current needs, the materials were updated between every round of the campaign. Unfortunately, the periodic educational communications were hampered by socioeconomic and political factors, cultural aspects, irregular reviews, and limited funds for production of materials. In addition, the lack of formative research on effective methods to conduct communication further compromised the dissemination of information.^9^^,^^13^

## Relevant changes

**Fig. 1:**
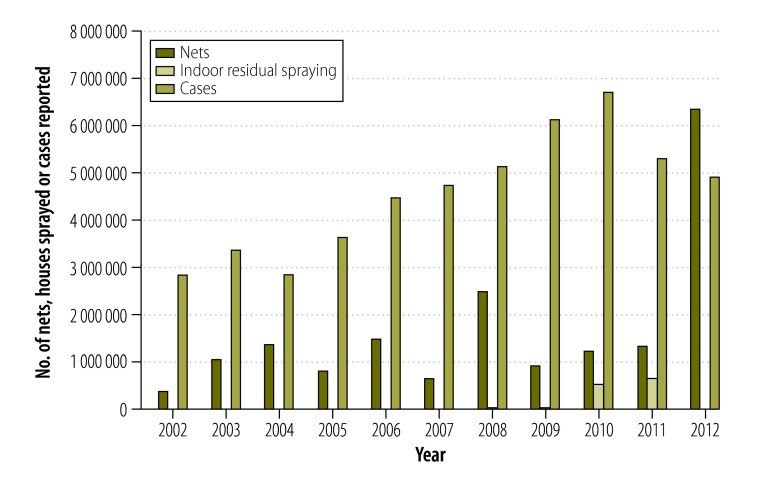
Numbers of malaria cases reported, long-lasting insecticide-treated nets distributed and coverage of indoor residual spraying, Malawi, 2002–2012

Between 2002 and 2012, the programme had distributed a total of 18 248 206 LLINs. The number of nets distributed per year increased from 372 991 in 2002 to 6 370 073 in 2012. For the same period, the coverage of at least one LLIN per household increased from 27% (3689/13 664) to 58% (1974/3404). Among children younger than five years, the use of LLINs increased from 15% (1581/10 539) to 56% (1397/2495), for pregnant women the use increased from 15% (211/1405) to 51% (127/250).^13^ In Nkhotakota, 28 227 structures were sprayed in 2007, which in 2008 increased to 42 044 and in 2009 to 56 729, protecting over 500 000 residents. Indoor spraying was scaled up to seven targeted districts in 2010 and 2011, with 527 372 and 653 592 structures sprayed, respectively.^10^ The annual malaria cases increased from 2 853 315 in 2002 to 6 748 535 in 2010. The indoor spraying in 2011 and the mass distribution of bednets in 2012 most likely reduced the number of cases to 4 922 596 in 2012 (Fig. 1).

In 2002, *A. arabiensis* was reported to be resistant to dichlorodiphenyltrichloroethane (DDT), but still susceptible to pyrethroids and organophosphates.^14^ However, in 2010, this species showed resistance to pyrethroids.^10^ Since 2010, *A. funestus* has also been shown to be resistant to pyrethroids and carbamates across the country, which led the programme to switch from pyrethroids to organophosphates for indoor spraying in 2011.^15^ The programme reduced the indoor spraying to only one district (Salima) in 2014 due to the exorbitant cost of organophosphate insecticides.^10^

## Challenges and lessons learnt

The vector control implementation faced several challenges (Table 1). The suboptimal LLIN coverage and use of nets coupled with alleged abuse of LLINs, are probably some of the explanations why the programme has not been as effective as anticipated. Another explanation could be that indoor spraying had to be scaled back due to insecticide resistance. The community sensitization, participation and ownership were sporadic resulting in refusals or low compliance with existing interventions.^9^^,^^13^ Supplementary interventions such as larval source management were not deployed due to lack of technical capacity. The technical design for the procurement of commodities and equipment was inadequate, including lack of consensus among stakeholders on using DDT for indoor spraying in selected areas with susceptible vectors and suboptimal information sharing regarding entomological resources. Limited partner support for vector control was a major constraint for funding leaving the Presidents Malaria Initiative and the Global Fund as the only funders amidst dwindling government support. By 2014, the integrated vector management approach was still not fully operationalized due to lack of funds, resulting in incomplete scale-up of interventions such as indoor spraying. Logistical problems including inadequate, unpredictable and late disbursement of government funds resulted in missed targets for indoor spraying in 2014.

**Table 1:** Challenges and risks encountered in integrated vector management and recommendations for improvement in Malawi, 2002–2012

Key element	Challenges and risks	Recommendation
Advocacy, social mobilization and legislation	Inconsistent community sensitization and mobilization, and low compliance, ownership and involvement in malaria vector control activities.	Conduct IEC/BCC campaigns, reinforce/mobilize community engagement and empowerment for participation and adopting relevant policies and ensuring legislation on vector control.
	Irregular IEC/BCC reviews and planning meetings, limited funds for production of IEC materials, IEC messages are available in limited languages. BCC has been focusing on creating awareness but not on behaviour change.	To create awareness and promote behaviour change, collaborate with the Health Education Unit, community radio stations and use the World Malaria Day and SADC malaria weeks.
	Low coverage and use of vector control interventions, occurrence of net misuse and low LLIN availability at ANC clinics and EPI services due to stock outs.	PMI to continue to fund national mass and print media campaigns to increase awareness, promote correct and nightly ITN use, and proper care and repair. Follow-up to ensure that ITN supplies are available at all facilities.
Collaboration within the health sector and with other sectors	Lack of consensus among stakeholders on the use of DDT for IRS, LSM and disposal mechanism for old LLINs.	Establish a national steering committee on IVM to ensure broader stakeholder engagement and strengthened intra- and inter-sectoral collaboration.
	Minimal collaboration between academic/scientific institutions and health ministry on entomological resources, including insecticide resistance and vector bionomics.	Establish a multidisciplinary decision-making body for entomological resources to make technical recommendations and give advice on vector control.
	Poor financial resources hindering the scale-up of some interventions.	Strengthen cooperation among stakeholders and advocate for political commitment to mobilize resources.
Integrated approach	Lack of consistence in timely deployment and scaling up of key vector control interventions to achieve universal coverage.	Update guidelines and policies for LLINs distribution and IRS implementation and ensure their universal access. Implementing evidence-based, cost-effective and sustainable interventions.
	Supplementary interventions, such as using larvicides and biological control, have not been implemented.	Develop requisite competences for deploying supplementary tools where feasible and mobilize the needed resources.
	Limited collaboration with other vector borne disease control programmes, such as lymphatic filariasis programme.	Strengthen collaboration with other vector control programmes and explore synergies for targeting different vectors.
Evidence-based decision-making	Absence of comprehensive malaria transmission data and minimal use of existing entomological data for decision-making for vector control.	Develop and implement a vector and epidemiological surveillance plan/system, establish sentinel sites and strengthen operational research and monitoring and evaluation to guide the scale-up of interventions.
	Emergence and spread of pyrethroid and carbamate resistance in malaria vectors have the potential to diminish the effectiveness of IRS and LLINs.	Conduct well-coordinated and intensive surveillance for insecticide resistance data to inform evidence-based response.
	Irregular insecticide resistance monitoring and management with limited documentation.	Develop and implement an insecticide resistance monitoring and management plan/strategy to facilitate vector resistance mapping and rational decision-making.
	Limited LLIN durability monitoring to inform replacement and innovation, nominal data preference of conical to rectangular-shaped nets that could affect use and retention of ITNs.	In Chikhwawa, ITN durability study suggests a high proportion of nets were heavily damaged and need replacement. Specific questions on net shape preference were included in the 2014 MIS to address concerns.
	There is inadequate information sharing between stakeholders for timely decision-making.	There is a need to establish a forum for research dissemination by all partners.
Capacity building	The number of entomologists is limited and only minimal capacities for entomological laboratory, infrastructure and logistics exist for vector management activities.	Build requisite institutional capacity for planning and implementing effective malaria vector control.
	Delays in disbursement of funding from the Global Fund, primarily due to issues related to government financial management systems for procurement and implementation.	PMI to continue to work with the National Malaria Control Programme to strengthen partnerships that exist between the programme and stakeholders around ITN procurement and distribution.
	Limited technical expertise on IVM. Inadequate experience in LSM and lack of compliance with vector control distribution guidelines.	Conduct certified courses on IVM and judicious use of pesticides.

ANC: antenatal clinic; BCC: behaviour change communication; DDT: dichlorodiphenyltrichloroethane; EPI: Expanded Programme on Immunization; IEC: information, education and communication; IRS: indoor residual spraying; ITN: insecticide treated nets; IVM: integrated vector management; LLIN: long-lasting insecticidal net; LSM: larval source management; MIS: Malaria Indicator Survey; PMI: President’s Malaria Initiative; SADC: Southern African Development Community.

The main lessons learnt are summarized in Box 1. While vector control seemed to be effective in 2011 and 2012 – by reducing the number of malaria cases – without investment in insecticide treated nets and indoor spraying, the malaria burden could worsen in the country. Accordingly, the programme developed a new integrated vector management strategy for 2015–2019, which established functional multidisciplinary coordination mechanisms and advocated for appropriate vector control interventions among key stakeholders.^12^ This strategy prioritizes: (i) adoption of a policy for universal coverage with LLINs – that is, to ensure that every sleeping space in every household is covered by an LLIN; (ii) indoor spraying with insecticides other than pyrethroids and carbamates, and that the selection of insecticides is based on epidemiological and entomological data; (iii) where feasible, supplementing indoor spraying and LLINs with focal larval source management, preferably using biological larvicides; and (iv) determining the diversity of vectors and establishing a rational insecticide resistance management strategy.^12^

Box 1 **Summary of main lessons learnt**

- In high-transmission areas, a single intervention approach for scaled-up malaria vector control may not have a substantial impact.
- For optimal use of resources, a well-coordinated integrated vector management strategy may offer greater benefits.
- For effective and sustainable vector control, an insecticide resistance monitoring and management plan involving all vector-control resources in the country is essential.

In Malawi, insecticides’ resistance limits the choice of insecticides for indoor spraying and threatens the continued effectiveness of insecticide treated nets.^14^^,^^15^ Strengthened entomological monitoring, including insecticide resistance management, would be critical for evidence-based, cost-effective, operationally scaled-up and sustainable vector control. Therefore, the programme aims to develop and implement a resistance monitoring and management plan and a vector surveillance plan.^12^

Despite the challenges, there is now a strong political commitment to vector control in Malawi. This includes wide-ranging partner support, increased social mobilization and advocacy by stakeholders, a high demand for LLINs by community members and the possibility of expanding indoor spraying through public–private sector partnerships, including consensus to pilot DDT-based indoor spraying by the programme in non-tobacco growing districts.^9^ However, successful integrated vector management demands for adequate financial and technical resources, strengthened operational research for evidence-based, effective targeting, deployment and monitoring of interventions. The programme will evaluate improvement in housing and introduction of larvivorous fish in earmarked areas amenable with the interventions, novel models of LLIN and repellents, and determine residual and/or outdoor transmission of malaria.^12^

In Malawi, a functioning vector control needs adequate financial resources and an integrated vector management strategy. Scale-up of vector control in similar settings will need to be carefully considered and adapted to the local situation in the context of the integrated vector management approach.
